# The marine triterpene glycoside frondoside A induces p53-independent apoptosis and inhibits autophagy in urothelial carcinoma cells

**DOI:** 10.1186/s12885-017-3085-z

**Published:** 2017-02-01

**Authors:** Sergey A. Dyshlovoy, Ramin Madanchi, Jessica Hauschild, Katharina Otte, Winfried H. Alsdorf, Udo Schumacher, Vladimir I. Kalinin, Alexandra S. Silchenko, Sergey A. Avilov, Friedemann Honecker, Valentin A. Stonik, Carsten Bokemeyer, Gunhild von Amsberg

**Affiliations:** 1 Laboratory of Experimental Oncology, Department of Oncology, Hematology and Bone Marrow Transplantation with Section Pneumology, University Medical Center Hamburg-Eppendorf, Martinistr. 52, Hamburg, 20246 Germany; 2 Laboratory of Marine Natural Products Chemistry, G.B. Elyakov Pacific Institute of Bioorganic Chemistry, Far-East Branch, Russian Academy of Sciences, Prospekt 100-let Vladivostoku 159, Vladivostok, 690022 Russian Federation; 3 School of Natural Sciences, Far Eastern Federal University, Sukhanova Street 8, Vladivostok, 690091 Russian Federation; 4 Institute of Anatomy and Experimental Morphology, University Cancer Center Hamburg, University Medical Center Hamburg-Eppendorf, Martinistr. 52, Hamburg, 20246 Germany; 5 Tumor and Breast Center ZeTuP St. Gallen, Rorschacher Strasse 150, St. Gallen, 9006 Switzerland

**Keywords:** Frondoside A, p53, Apoptosis, Autophagy, Urothelial carcinoma, Marine natural compounds

## Abstract

**Background:**

Advanced urothelial carcinomas represent a considerable clinical challenge as they are difficult to treat. Platinum-based combination regimens obtain response rates ranging from 40 to 70% in first-line therapy of advanced urothelial carcinoma. In the majority of cases, however, the duration of these responses is limited, and when progression occurs, the outcome is generally poor. Therefore, novel therapeutic strategies are urgently needed. The purpose of the current research is to investigate the anticancer effects and the mode of action of the marine triterpene glycoside frondoside A in p53-wild type and p53-deficient human urothelial carcinoma cells.

**Methods:**

Activity of frondoside A was examined in the human urothelial carcinoma cell lines RT112, RT4, HT-1197, TCC-SUP, T-24, and 486p. Effects of frondoside A on cell viability, either alone or in combination with standard cytotoxic agents were investigated, and synergistic effects were analyzed. Pro-apoptotic activity was assessed by Western blotting and FACS, alone and in combination with a caspases-inhibitor. The impact of functional p53 was investigated by siRNA gene silencing and the p53 inhibitor pifithrin-α. Effects on autophagy were studied using LC3B-I/II and SQSTM/p62 as markers. The unpaired Student’s *t*-test was used for comparison of the data sets.

**Results:**

Frondoside A shows high cytotoxicity in urothelial carcinoma cells with IC_50s_ ranging from 0.55 to 2.33 μM while higher concentrations of cisplatin are required for comparable effects (IC_50_ = 2.03 ~ 5.88 μM). Induction of apoptosis by frondoside A was associated with the regulation of several pro-apoptotic factors, like caspase-3, -8, and -9, PARP, Bax, p21, DNA fragmentation, and externalization of phosphatidylserine. Remarkably, inhibition of p53 by gene silencing or pifithrin-α pretreatment, as well as caspase inhibition, did not suppress apoptotic activity of frondoside A, while cisplatin activity, in contrast, was significantly decreased. Frondoside A inhibited pro-survival autophagy, a known mechanism of drug resistance in urothelial carcinoma and showed synergistic activity with cisplatin and gemcitabine.

**Conclusions:**

A unique combination of properties makes marine compound frondoside A a promising candidate for the treatment of human urothelial carcinomas.

**Electronic supplementary material:**

The online version of this article (doi:10.1186/s12885-017-3085-z) contains supplementary material, which is available to authorized users.

## Background

Advanced urothelial carcinomas (UCs) represent a considerable clinical challenge as they are difficult to treat. Platinum-based combination regimens obtain response rates ranging from 40 to 70% in first-line therapy of advanced urothelial carcinoma (UC). In the majority of cases, however, the duration of these responses is limited, and when progression occurs, the outcome is generally poor [1]. So far, the results of single agents or combinations administered in the salvage setting have been rather disappointing [2]. In Europe, vinflunine is the only approved second-line therapy to date, and until recently, no treatment regimen was officially recommended in the USA for salvage therapy [3]. However, on May 18th 2016, the PD-L1 inhibitor atezolizumab was approved by the U.S. Food and Drug Administration (FDA) for the treatment of patients with locally advanced or metastatic UC who experienced disease progression during or following platinum-containing chemotherapy. In fact, atezolizumab achieved objective response rates of 26% (95% CI 18–36) in the IC2/3 group, 18% (13–24) in the IC1/2/3 group, and 15% (11–19) in all treated patients of a phase II clinical trial (IMvigor210) with some long-term responses lasting for more than 12 months [4, 5]. Nevertheless, patients, especially without PD-L1 expression, may not significantly benefit from this immune check-point inhibition, emphasizing an unmet need for novel therapeutic strategies [4].

p53 deficiency has been detected in more than 60% of UC [6], and has been associated with aggressive biology [7, 8]. In addition, it has been speculated that it could serve as a prognostic marker for the response of UC to systemic treatment [7, 8]. Furthermore, p53 deficiency or inactivation is a known mechanism of drug resistance in human malignancies [9].

Another factor playing a role in chemotherapy resistance is autophagy [10, 11]. Macroautophagy (referred as autophagy) is a basic cellular catabolic process which leads to selective or non-selective degradation of proteins and organelles by the lysosome system [12]. It includes the formation of double-membrane vesicles called autophagosomes, which fuse with lysosomes, resulting in the degradation and recycling of sequestered contents [12]. Most of anticancer drug treatment-induced autophagy was identified to have pro-survival properties and therefore, this process is believed to be one of the key mechanisms of drug resistance [10, 11]. Autophagy allows cancer cells to use their entire resources to survive diverse apoptosis inducing signals, including chemotherapy-induced apoptosis [11]. Recently, autophagy has been reported to be an important drug resistance and pro-survival mechanism in UC [10, 13]. Therefore, pharmacological inhibitors of autophagy may prevent development of resistance and enhance cytotoxic activity of known anticancer drugs [10, 11].

Frondoside A (FrA) is a natural bioactive compound, initially isolated from the sea cucumber *Cucumaria frondosa* [14]. Recent studies revealed promising anticancer activity of FrA in vitro and in vivo, which is exerted through its pro-apoptotic, antimetastatic, and immunostimulatory activity (for review see [15, 16]). Remarkably, we were able to demonstrate that FrA inhibits pro-survival autophagy in prostate cancer cell lines resistant to standard therapies and induces apoptosis [17]. However, to date, no data are available on the activity of FrA in human UC.

In this study we examined the effect of the triterpene glycoside FrA in human UC cells bearing either wild-type or mutant p53. We explored the relevance of p53 for the anti-cancer effect of the marine natural compound, as well as the effect of FrA on autophagy in UC cells.

## Methods

### Reagents and antibodies

The marine triterpene glycoside frondoside A (FrA) was isolated from the sea cucumber *Cucumaria okhotensis* as previously described [18]. The purity of the individual compound was verified by HPLC, ^1^H and ^13^C NMR spectroscopy. Other reagents and antibodies are listed in Additional file 1.

### Cell lines and culture conditions

The human urothelial cancer cell lines RT4 (p53 wild type), HT-1197 (p53 wild type), TCC-SUP (mutant p53), T-24 (mutant p53), were purchased from ATCC (Manassas, VA, USA) [19]. RT112 (p53 wild type) cell line was purchased from DSMZ (Braunschweig, Germany) [19]. 486p cell line (unknown p53 mutational status) was previously generated and characterized by Elliott et al. from grade IV TCC of the bladder metastatic to a supraclavicular node of 61-year-old white male patient [20, 21]; ethics and consent statements can be found in the corresponding references [20, 21]. 486p cells were kindly provided by the Urology department of University Medical Center Hamburg-Eppendorf, Hamburg, Germany. Culture conditions are described in the Additional file 1.

### In vitro cell viability assays

Cytotoxicity profiles of single compounds and drug combinations were evaluated by MTT or trypan blue-based viability assays as described previously [22]. The duration of treatment was 48 h, unless otherwise stated.

### Examination of synergistic/antagonistic effects of drug combinations

Determination of synergistic, antagonistic, or additive effects of compounds used in combination assays was performed using the Chou-Talalay method as previously described [23]. The combinational index (CI) was calculated with the CompuSyn v.1.0. Software (ComboSyn, Inc., Paramus, NJ, USA). Fa (fraction affected) is defined as the non-survival fraction at a certain dose of compounds or their combinations. Synergism is defined as a CI < 0.85, whereas antagonism has a CI > 1.2. A CI of 0.85 to 1.2 is considered an additive effect.

### Detection of apoptotic cells by annexin-V-FITC/PI double staining

Induction of apoptosis was examined by FACS analysis with an annexin-V-FITC and propidium iodide (PI) double staining. The experiment was performed as previously described with slight modifications [24]. In brief, cells were pre-incubated overnight in 6-well plates (0.2 × 10^6^ cells/well), pretreated for 1 h with the medium (1 mL/well) with or without addition of the caspase inhibitor zVAD (100 μM). The compound of interest was then added and the cells were incubated for additional 48 h. After treatment, cells were harvested with a trypsin solution, stained, and analyzed using a FACS Calibur (BD Bioscience) and BD Bioscience Cell Quest Pro software (BD Bioscience).

### Cell cycle and DNA fragmentation analysis

The cell cycle distribution was analyzed by flow cytometry using PI staining as described before [22]. In brief, cells were pre-incubated overnight in 6-well plates (0.2 × 10^6^ cells/well) and treated with FrA. After 48 h of treatment, cells were trypsinized, fixed with 70% EtOH/H_2_O (v/v), stained, and analyzed. The results were generated and quantitatively analyzed using a FACS Calibur as above and BD Bioscience Cell Quest Pro software.

### Western blotting

Preparation of protein extracts and Western blotting was performed as described previously [23]. In brief 1 × 10^6^ cells/well were seeded in Petri dishes (ø 10 cm TC Dish (Sarstedt, Numbrecht, Germany) 10 mL/dish), incubated overnight and treated with drugs for 48 h in 10 mL/dish. Cells were harvested using a cell scraper, washed, and lysed. Lysates were frozen overnight at −20 °C and then centrifuged. Protein concentration in the supernatants was determined by Bradford assay. Total protein extracts (20–30 μg/sample) were subjected to electrophoresis in SDS-polyacrylamide gels at 120 V, and transferred from gel to a 0.2 μm pore PVDF membrane. The membrane was blocked and incubated with the primary and secondary antibodies according to the manufacturers’ protocol (for antibodies used, see Additional file 1: Table S1). Signals were detected using the ECL chemiluminescence system (Thermo Scientific, Rockford, IL, USA) according to the manufacturer’s protocol.

### Silencing of p53 by siRNA transfection

Silencing of p53 gene was performed using siRNA transfection technique and Lipofectamine® RNAiMAX Transfection Reagent (Invitrogen, UK). RT112 cells were pre-incubated overnight in 6-well plates (1 × 10^5^ cells/well in 2 mL) in antibiotics-free RPMI media. The solutions (a) and (b) were prepared: (a) 20 μL of 100 pmol/μL siRNA (total amount 2 nmol) + 230 μL of Opti-MEM media; (b) 7.5 μL of Lipofectamine® RNAiMAX Transfection Reagent + 242.5 μL of Opti-MEM media. The solutions were incubated for 5 min, mixed and further incubated for 20 min. The media in the wells were replaced with 2 mL of fresh antibiotics-free RPMI media and 0.5 mL of (a) + (b) mixture were added to each well by dropping. After 72 h of incubation the media was aspirated, cells were washed PBS, and fresh antibiotics-free RPMI media (drug-containing or drug-free) was added (2 mL/well). Then the cells were either immediately harvested for Western blotting analysis or incubated for 48 h and analyzed by FACS.

Duplexed siRNA were purchased from Eurofins Genomics (Ebersberg, Germany). The gene target sequences (5′ → 3′) are: p53 siRNA (NM_000546_Val): GACUCCAGU GGUAAUCUAC(dTdT); scrambled siRNA (Non Specific Control 47% GC): AGGUAGUGUAAUCGCCUUG(dTdT).

### Immunofluorescence analyses

The experiments were performed as described before [17]. In brief, RT112 cells (5 × 10^4^ cells/chamber) were treated for 48 h with investigated drugs, fixed and permeabilized. Next, cells were stained with anti-LC3B-I/II antibody overnight at 4 °C followed by incubation with secondary anti-rabbit Alexa Fluor 488-conjugated antibody. Samples were washed and covered with DAPI-based ProLong® Gold reagent (Life Technologies) and directly analyzed with AxioScope.A1 (Carl Zeiss) microscope with the AxioVision40 V4.8 software (Carl Zeiss Imaging Solutions).

### Statistical analyses

Statistical analyses were performed using GraphPad Prism software v. 5.01 (GraphPad Prism software Inc., La Jolla, CA, USA). Data are presented as mean ± SEM (standard error of mean). All experiments were performed in triplicates, and repeated at least three times. The unpaired Student’s *t*-test was used for comparison of two groups. Statistical significance was labeled as: *p < 0.05, **p < 0.01, ***p < 0.001.

## Results

### FrA reduces urothelial carcinoma cell viability

**Fig. 1:**
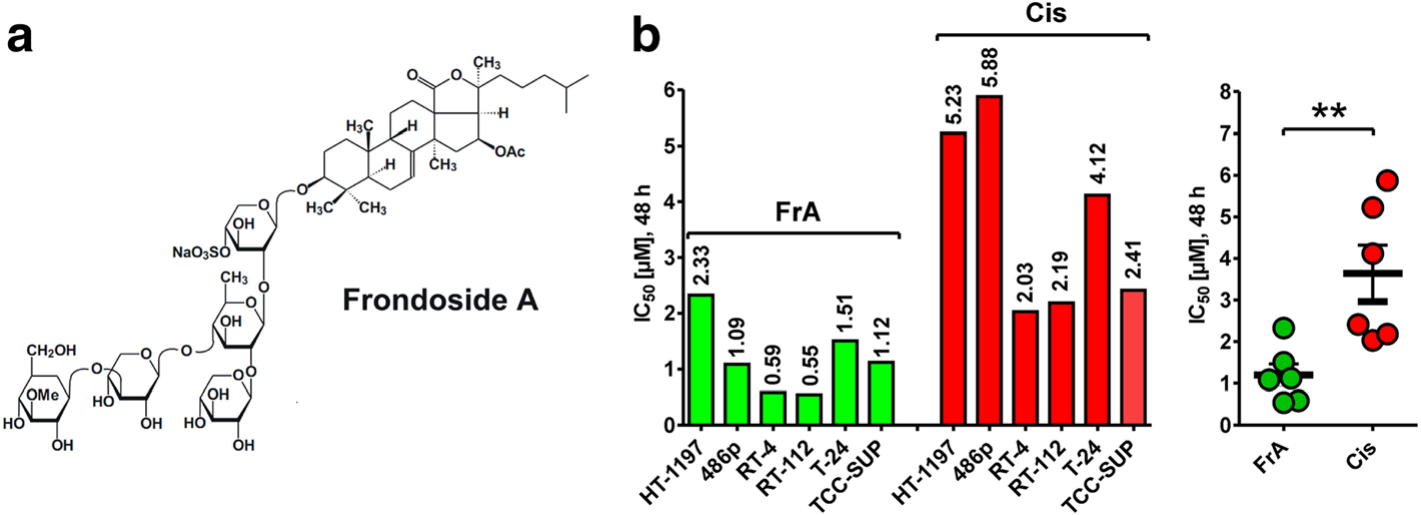
Structure and effect of FrA on the viability of human urothelial cancer cells. **a** Structure of frondoside A (FrA). **b** Cytotoxicity of FrA, determined with a trypan blue-based viability assay. Cells were treated with FrA or Cis for 48 h

Frondoside A (FrA, Fig. 1a) exerted cytotoxic activity in all human UC cell lines tested, with IC_50_s ranging from 0.55 to 2.33 μM. Remarkably, cisplatin (Cis) used as a reference drug was less effective in the same assays, having 2–6 fold higher IC_50_s (Fig. 1b). The urinary bladder transitional cell carcinoma cell line RT112 cells showed highest sensitivity to FrA, and was chosen as a model to further explore the molecular mechanisms of anticancer action of this marine natural compound.

### FrA induces apoptosis in urothelial carcinoma cells

**Fig. 2:**
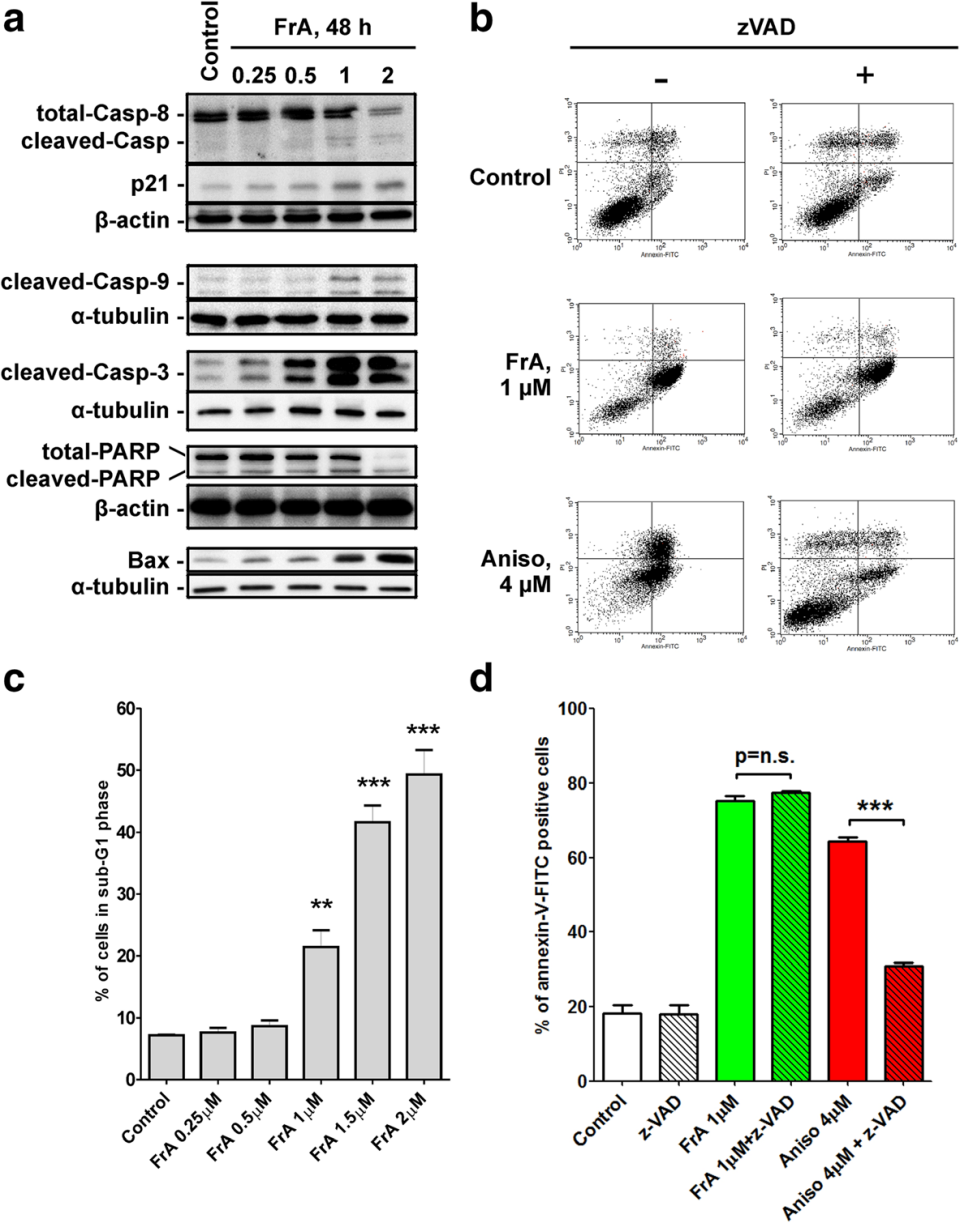
Induction of caspase-independent apoptosis in FrA-treated cells. **a** Western blotting analysis of protein extracts of RT112 cells treated with FrA for 48 h. **b** Cell cycle analysis of RT112 cells treated with FrA for 48 h. Apoptotic cells are detectable as a sub-G1 population. Cell cycle phase distribution was quantified using the Cell Quest Pro software. **c**, **d** Flow cytometry analysis of RT112 cells treated with FrA using an annexin-V-FITC/PI double staining (**c**) and quantification of positive cells (**d**). Cells were pretreated with 100 μM of the pan-caspase inhibitor zVAD for 1 h and then treated with indicated concentrations of FrA or with anisomycin (Aniso, positive control) for 48 h. Apoptotic cells appearing in the right lower and upper quadrants were quantified using the Cell Quest Pro software

Next, we examined the effect of FrA on the induction of apoptosis in human UC cells. Hallmarks of apoptosis, including dose-dependent caspase- and PARP-cleavage (Fig. 2a), DNA fragmentation (Fig. 2b), as well as phosphatidylserine externalization (Fig. 2c), were observed in FrA-treated RT112 cells. In addition, the effect of FrA on several pro- and anti-apoptotic proteins was examined by Western blotting. The upregulation of pro-apoptotic Bax and p21 was observed in FrA-treated RT112 cells (Fig. 2a), while no alterations of p-Akt, p53, Bad, Pak1, survivin, and Bcl-2 were found (data not shown). FACS analysis did not reveal any significant effect of FrA on cell cycle phase distribution of human UC cells (data not shown).

### FrA-induced apoptosis is caspase- and p53-independent

Various cytotoxic anti-cancer therapies act through pro-apoptotic caspases- and/or p53-related pathways. However, these processes are often defected in human cancer cells, which may result in chemotherapy resistance [9, 25]. Therefore, we investigated the role of these two pathways in FrA-induced apoptosis. We could show that FrA induces dose-dependent caspase-3, -8, and -9 cleavage in RT112 cells (Fig. 2a). However, the inhibition of caspases activity by pretreatment with the pan-caspase inhibitor zVAD did not decrease the apoptotic cells rate (Fig. 2c, d). At the same time, the pro-apoptotic effect of anisomycin–a well characterized inducer of classical apoptosis–was significantly inhibited by the zVAD (Fig. 2d). Based on these results, we conclude that FrA is able to induce apoptosis in UC cells independently of caspase activity. The observed caspase cleavage is probably an unspecific effect, which can occur secondary to other FrA-induced cytotoxic events.

**Fig. 3:**
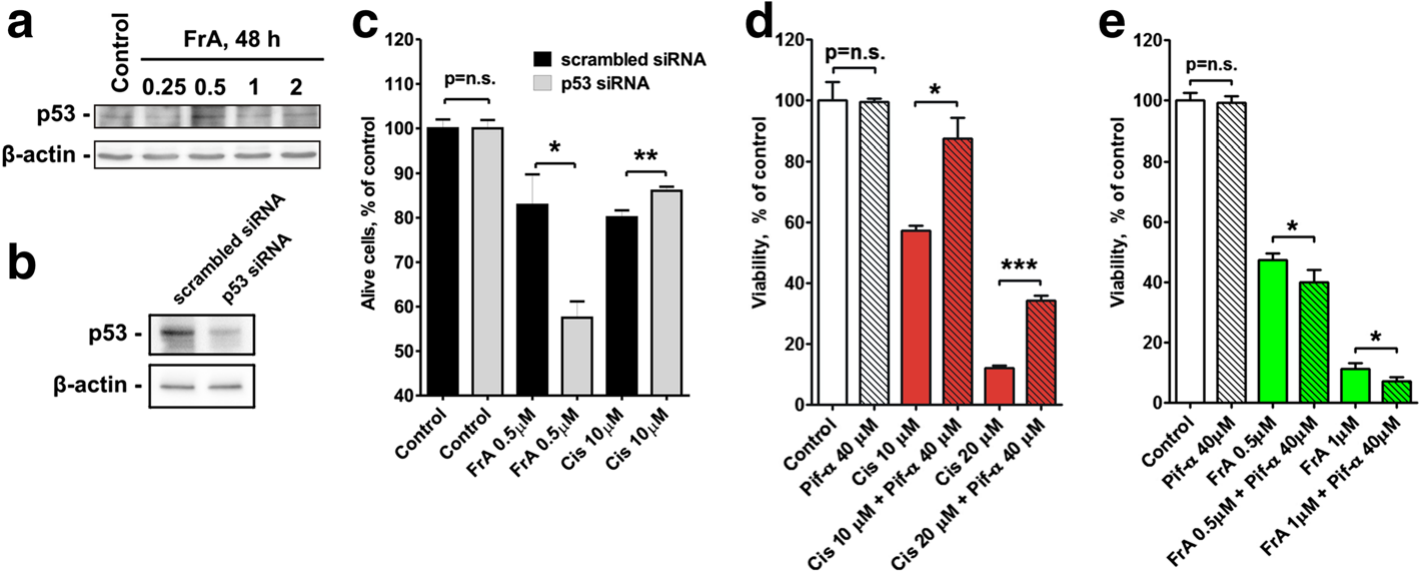
Effect of p53 silencing on cytotoxicity of FrA. **a**, **b** Western blotting analysis of p53 expression in RT112 cells treated with FrA for 48 h (**a**) or transfected with p53 siRNA versus cells transfected with scrambled siRNA (**b**). **c** Viability of transfected cells treated with FrA or Cis for 48 h. Cell viability was analyzed by flow cytometry analysis using annexin-V-FITC/PI double staining. Viable cells appearing in the lower left quadrant were quantified using the Cell Quest Pro software. **d**, **e** Effect of pifithrin-α (Pif-α)–an inhibitor of p53 activity–on the cytotoxic activity of FrA and Cis in nontransfected RT112 cells. Cells were pretreated with 40 μM of Pif-α for 30 min and then cotreated with FrA (**d**) or Cis (**e**) for 48 h. Cell viability was determined using MTT-assay

RT112 cells are known to harbor wild-type p53 gene [19]. FrA did not alter the total level of p53 of RT112 cells (Fig. 3a). Silencing of the p53 gene expression using specific siRNA resulted in a substantial reduction of p53 protein level in RT112 cells (Fig. 3b). Remarkably, the cytotoxic effect of cisplatin was significantly decreased in the cells with reduced p53 level, while no inhibition of FrA efficacy was observed (Fig. 3c). In line with this finding, pretreatment with pifithrin-α–a chemical inhibitor of p53 activity–suppressed cytotoxicity of cisplatin (Fig. 3d), but not the activity of FrA (Fig. 3e) suggesting that FrA remains active in human cancer cells bearing mutated non-functional p53.

### FrA affects MAPK in RT112 cells

**Fig. 4:**
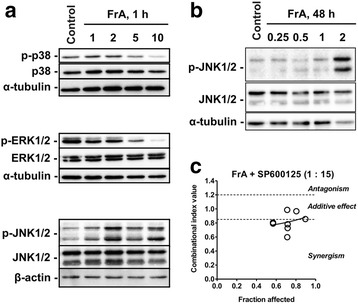
Effect of FrA on mitogen-activated protein kinases (MAPK). **a**, **b** RT112 cells were treated with FrA for 1 h (**a**) or 48 h (**b**), and then protein extracts were analyzed by Western blotting. **c** Effect of SP600125 (a specific JNK1/2 inhibitor) on the survival of RT112 cells treated with FrA. Drugs were combined in the constant molar ratio C(FrA) : C(SP600125) = 1 : 15, and cells were cotreated with the individual drugs or their combination for 48 h. The data were generated using trypan blue-based viability assay. Cells were pretreated with SP600125 in 50 μL/well culture media for 1 h. The combinational index (CI) was calculated with the CompuSyn v.1.0. software

Mitogen activated protein kinases (MAPK) can be involved in both proliferative or pro-apoptotic mechanisms in human UC [26, 27]. Therefore, we evaluated the effect of FrA on MAPK in RT112 cells. After short-term treatment (1 h), FrA lead to suppression of p38 and ERK1/2 phosphorylation (at the FrA concentrations ≥ 5 μM), but activated JNK1/2 (Fig. 4a). Activation of JNK1/2 was also observed after long-term treatment with FrA (48 h, Fig. 4b). To further explore the role of JNK1/2 activation in response to FrA treatment, we investigated the effect of the well-established JNK1/2 inhibitor SP600125 on FrA-mediated cytotoxicity. Combining FrA and SP600125 clearly showed synergistic cytotoxic effects in MTT-based Chou-Talalay assays (Fig. 4c), indicating a prosurvival role of JNK1/2 activation in UC cells following FrA treatment.

### Autophagy

**Fig. 5:**
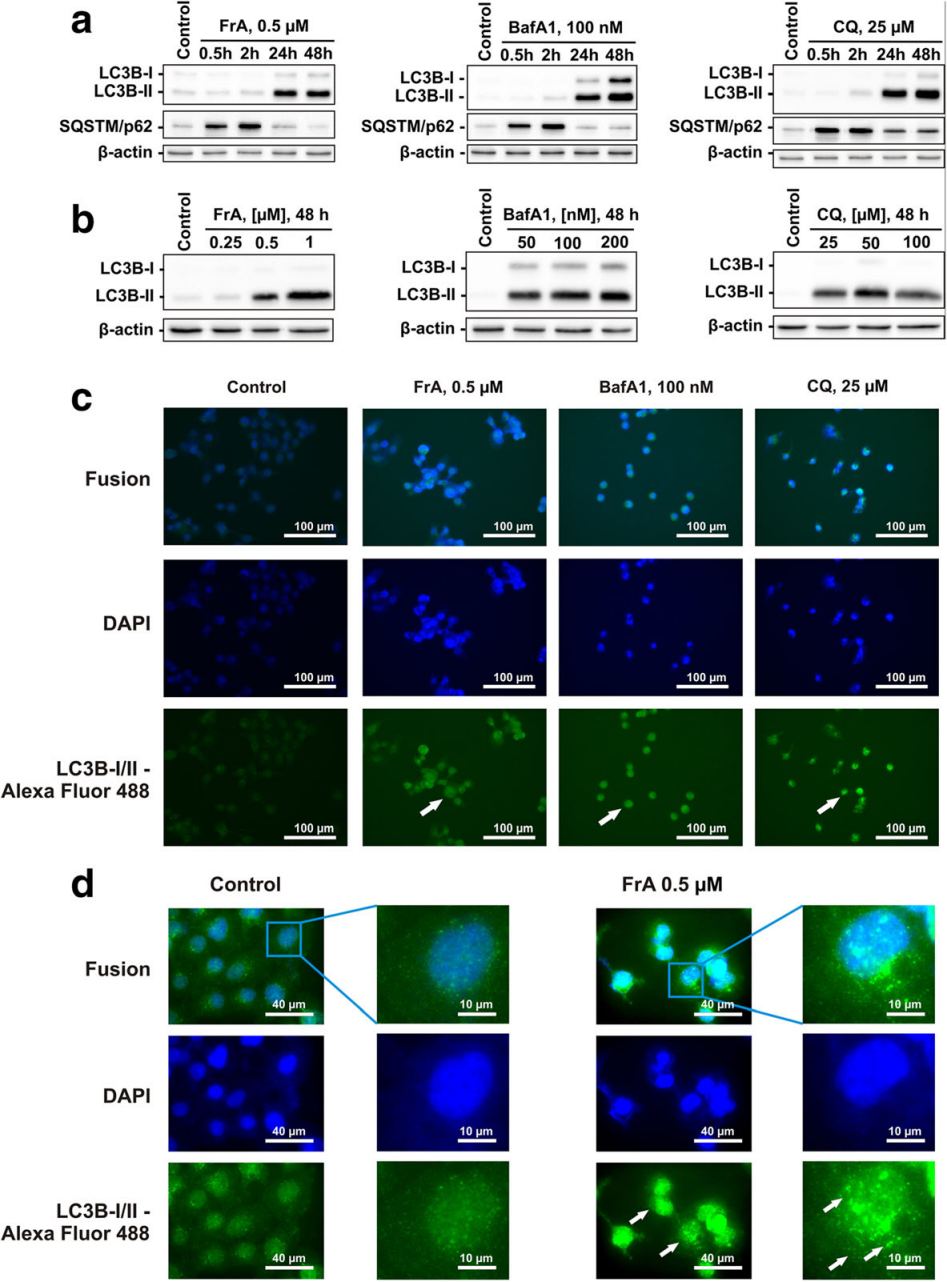
Inhibition of autophagy in urothelial cancer cells under FrA-treatment. **a**, **b** Time- (**a**) and dose-dependent (**b**) effects of FrA, bafilomycin A1 (BafA1), and chloroquine (CQ) on levels of LC3B-I/II and SQSTM/p62 in RT112 cells. Cells were treated for 0.5–48 h (**a**) or for 48 h (**b**), followed by protein extraction and analysis by Western blotting. The established autophagy inhibitors BafA1 and CQ were used as positive controls. **c**, **d** Accumulation of autophagosomes/autolysosomes. Cells were treated with the indicated concentrations of FrA, BafA1, and CQ for 48 h, fixed, permeabilized, and incubated with the anti-LC3B-I/II antibody, followed by treatment with Alexa Fluor 488-conjugated secondary antibody. The pictures were made at × 400 (**c**) or × 1000 (**d**) magnification. LC3B-I/II-positive organelles (autophagosomes/autolysosomes) appearing as dots are indicated by *arrows*

Induction of pro-survival autophagy by different anti-cancer agents is a recently characterized phenomenon in different human cancers, including UC [10, 11, 28]. Of note, pro-surival autophagy was identified as one of the main mechanisms of drug-resistance in UC [10, 11, 13, 28]. LC3 (isoforms LC3B-I and LC3B-II) and p62 (also known as SQSTM1) proteins are major effectors of this process and therefore are often used as autophagy alteration markers [29]. During autophagy, LC3-I converts to LC3-II, which is required for the autophagosome membrane formation. p62 is an autophagosome cargo protein which binds other proteins for selective autophagy [29]. Accumulation of LC3-I/II and p62 as well as accumulation of LC3-positive organelles (autophagosomes) indicate inhibition of autophagy [29]. We could demonstrate that FrA induces time- and dose-dependent accumulation of these autophagy-related proteins (Fig. 5a, b). Interestingly, in RT112 cells, the maximal p62 protein level was observed after 2 h of treatment, whereas maximal LC3B-II levels were detected after 48 h (Fig. 5a, b), elucidating the kinetics of autophagy in UC cells. Additionally, increased LC3B-I/II-immunostaining of RT112 cells treated with FrA (Fig. 5c) was found being a result of a treatment-induced accumulation of autophagosomes (Fig. 5d).

Finally, similar profiles were observed for cells treated with the well-established autophagy inhibitors BafA1 and CQ when compared to FrA (Fig. 5a–c), indicating comparable molecular effects of these three compounds, i.e. inhibition of autophagy.

### FrA enhances cytotoxic effects of cisplatin and gemcitabine

**Fig. 6:**
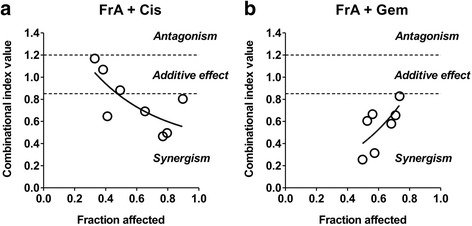
Effect of FrA in combination with cisplatin and gemcitabine. Cells were cotreated with different concentrations of the single substances or their combination for 48 h at the constant molar ratios C(FrA) : C(Cis) = 1 : 4 (**a**) and C(FrA) : C(Gem) = 1 : 0.25 (**b**). The combinational index (CI) values were calculated with CompuSyn software. The viability was examined using a trypan blue-based viability assay

The effect of FrA was evaluated in combination with two standard chemotherapeutic agents frequently applied in advanced UC, namely cisplatin and gemcitabine. Remarkably, FrA was strongly synergistic in combination with both drugs in RT112 cells (Fig. 6a, b).

## Discussion

Cisplatin based combination therapy is the standard of care for first line therapy of advanced or metastasized UC. However, despite initial response rates of 40–70%, relapses occur in the majority of patients due to the development of resistance [1, 10, 30]. Among others, overexpression of ERCC1, Nrf2, CTR1/2, hENT1 and BRCA1, expression of specific miRNA, epithelial-mesenchymal transition, loss of p53 function, and pro-survival autophagy were identified as potential mechanisms of resistance [10, 30].

In this study, we investigated the anticancer activity of the marine natural compound FrA in human UC cells. FrA decreased the viability of UC cells more effectively than cisplatin at equimolar concentrations. The marine compound exerted its cytotoxic action through the induction of apoptosis. This process was associated with the alteration of several pro-apoptotic factors, like cleaved caspases-3, -8, and -9, cleaved PARP, Bax and p21, leading to dose-dependent DNA fragmentation and phosphatidylserine externalization. In addition, FrA activated JNK1/2, while p38 and ERK1/2 were inhibited. JNK1/2 may have either pro- or anti-apoptotic functions, depending on cell type, nature of the death stimulus and other factors [31]. As inhibition of JNK1/2 resulted in a more pronounced cytotoxic effect of FrA, JNK1/2 activation may partly antagonize FrA mediated effects in UC cells. Combining FrA with a JNK1/2 inhibitor might increase the efficacy of the marine compound. These observations, however, require further investigations.

Caspase-dependent apoptosis pathways are known to be often nonfunctional in human cancer cells. Overexpression of endogenous caspase inhibitors, mutations in caspase genes and genes coding up- or downstream molecules, as well as low expression of these genes were found to be causative [25]. In addition, more than 50% of all human tumors (and >60% of UC neoplasms [6]) harbor mutant p53 with abrogated tumor suppressive function. This has often been associated with increased tumor progression and negative treatment outcome [7–9]. Thus, compounds which are able to induce apoptosis independently of caspase- and p53-activity are potentially of high clinical impact [9]. Interestingly, FrA mediated induction of apoptosis in human UC cells did not require active caspases, although the upregulation of active caspases was observed in the treated cells. Therefore, we assume that caspase activation is most probably a secondary event, triggered by programmed death of cancer cells in a caspase-independent manner [32, 33]. More importantly, using two different independent methods, namely p53 gene silencing and inhibition of p53 activity by pifithrin-α, we have demonstrated that FrA-induced apoptosis could not be abolished by p53 alterations. In line with these results, FrA was active in UC cells bearing both wild-type (RT112, RT4, and HT-1197 cells) and mutant (T-24 and TCC-SUP cells) p53 [19]. Interestingly, FrA appeared to be even slightly more effective in RT112 cells with silenced or inhibited p53. In contrast, in both experiments cisplatin was significantly less active in cells with either silenced or inhibited p53, demonstrating a p53-dependence of cytotoxicity of this classical anti-cancer agent. In the past decade, there are different hints that p53 engages powerful pro-survival pathways along with its tumor suppressive function [34]. This effect strongly depends on the cell type, the specific stimulus and its severity. In fact, unincisive stimuli can induce slight and temporary p53-dependent cell cycle arrest leading to DNA reparation. An explanation for the increase of FrA cytotoxicity in p53-deficient/suppressed RT112 cells may be a very low interaction of FrA with the DNA damaging system, leading to the activation of the pro-survival function of p53 (which is also in line with the observed slight activation of p21 expression–one of the best studied targets of p53). In contrast, cisplatin directly targets DNA by the induction of crosslinks (which is usually associated with the strong activation of p21 expression) [35], and therefore can be considered a “strong stimulus” activating the pro-apoptotic functions of p53. However, this issue requires further investigations.

Caspase-independent cell death (CICD) can be exerted through different mechanisms including death receptor-induced necroptosis as well as mitochondrial, lysosomal, or endoplasmic reticulum stress [33, 36, 37]. While the first mechanism requires the death ligand presence, the latter three are often trigged by small molecules which result in a mechanical damage of the respective cellular structures [36, 38]. Different proteins which are released during these events lead to the caspase-independent apoptosis not requiring active p53 protein. Thus, mitochondrial outer membrane permeabilization (MOMP) can cause the release of HtrA2/Omi, endonuclease G and AIF, while lysosomal membrane permeabilization (LMP) leads to the release of cathapsins, and endoplasmic reticulum (ER) stress–to calcium release, consequently leading to the activation of nonlysosomal cysteine proteases calpains [33, 36]. One of these mechanisms may explain caspase- and p53-independent character of FrA-induced apoptosis in UC cells. However, further investigations are required to prove these assumptions.

Recently, autophagy has been reported to be an important drug-resistance and pro-survival mechanism in UC [10, 13, 28]. Indeed, inhibition of basal levels of autophagy lead to UC cell death and re-sensitized cancer cells to chemotherapy [10, 13]. Here, we could demonstrate that FrA is capable to induce apoptosis and–unlike other cytotoxic compounds–simultaneously inhibit autophagy in UC cells.

Finally, FrA revealed a strong synergistic effect when combined with cisplatin and gemcitabine in RT112 cells. This effect may at least in part be explained by the ability of FrA to inhibit pro-survival autophagy, as similar effects were observed previously when combining cisplatin with autophagy inhibition with in UC cells [10].

In vivo studies are an essential step for the further preclinical and clinical development of anticancer agents. Recently, it has been shown that daily intravenous (i.v.) injections are the best way of FrA administration providing the highest values of maximum plasma concentration of this drug [39]. Therefore, it is highly recommended to use the i.v. administration for future in vivo studies of FrA in models of human UC.

## Conclusions

In conclusion, the marine triterpene glycoside frondoside A (FrA) is an interesting compound revealing high efficacy in human UC cells. A unique combination of properties including (i) induction of apoptosis not requiring active caspases and p53, (ii) inhibition of pro-survival autophagy and (iii) increased activity when combined with classical cytotoxic agents makes the FrA a very promising candidate for the treatment of UC.

## Additional file

Additional file 1: Table S1. Supplementary methods section. Additional information to the Materials section listing and describing the reagents and antibodies used as well as the cell culture conditions. (DOCX 25 kb)

## Abbrevations

Aniso: Anisomycin
BafA1: Bafilomycin A1
CI: Combinational index
Cis: Cisplatin
CQ: Chloroquine
FrA: Frondoside A
Gem: Gemcitabine
Pif-α: Pifithrin-α
UC: Urothelial carcinoma
zVAD: z-VAD(OMe)-fmk
