# Supplementary material for: The marine triterpene glycoside frondoside A induces p53-independent apoptosis and inhibits autophagy in urothelial carcinoma cells
Source: BMC Cancer. 2017 Feb 1;17:93. doi: 10.1186/s12885-017-3085-z (PMC5286817; doi:10.1186/s12885-017-3085-z)
Supplement: Additional file 1: Table S1. — Supplementary methods section. Additional information to the Materials section listing and describing the reagents and antibodies used as well as the cell culture conditions. (DOCX 25 kb) [file 12885_2017_3085_MOESM1_ESM.docx]

Supplementary methods section

**The marine triterpene glycoside frondoside A induces p53-independent apoptosis and inhibits autophagy in urothelial carcinoma cells**

Sergey A. Dyshlovoy^1,2,3,*^, Ramin Madanchi^1,*^, Jessica Hauschild^1^, Katharina Otte^1^, Winfried H. Alsdorf^1^, Udo Schumacher^4^, Vladimir I. Kalinin^2^, Alexandra S. Silchenko^2^, Sergey A. Avilov^2^, Friedemann Honecker^1,5^, Valentin A. Stonik^2^, Carsten Bokemeyer^1^, Gunhild von Amsberg^1^

^1^ Laboratory of Experimental Oncology, Department of Oncology, Hematology and Bone Marrow Transplantation with Section Pneumology, Hubertus Wald-Tumorzentrum, University Medical Center Hamburg-Eppendorf, Martinistr. 52, 20246 Hamburg, Germany; [dyshlovoy@gmail.com](mailto:dyshlovoy@gmail.com) (S.A.D.); [j.hauschild@uke.de](mailto:j.hauschild@uke.de) (J.H.); [k.otte@uke.de](mailto:k.otte@uke.de) (K.O.); [c.bokemeyer@uke.de](mailto:c.bokemeyer@uke.de) (C.B.); [ramin.madanchi@stud.uke.uni-hamburg.de](mailto:ramin.madanchi@stud.uke.uni-hamburg.de) (R.M.); [w.alsdorf@uke.de](mailto:w.alsdorf@uke.de) (W.H.A.) ; [friedemann.honecker@zetup.ch](mailto:friedemann.honecker@zetup.ch) (F.H.); [g.von-amsberg@uke.de](mailto:g.von-amsberg@uke.de) (G. v. A).

^2^ Laboratory of Marine Natural Products Chemistry, G.B. Elyakov Paciﬁc Institute of Bioorganic Chemistry, Far-East Branch, Russian Academy of Sciences, Prospekt 100-let Vladivostoku 159, 690022 Vladivostok, Russian Federation; [kalininv@piboc.dvo.ru](mailto:kalininv@piboc.dvo.ru) (V.I.K.); [alex@piboc.dvo.ru](mailto:alex@piboc.dvo.ru) (A.S.S.); [avilov@piboc.dvo.ru](mailto:avilov@piboc.dvo.ru) (S.A.A.); [stonik@piboc.dvo.ru](mailto:stonik@piboc.dvo.ru) (V.A.S.)

^3^ School of Natural Sciences, Far East Federal University, Sukhanova Street 8, 690091 Vladivostok, Russian Federation

^4^ Institute of Anatomy and Experimental Morphology, University Cancer Center Hamburg, University Medical Center Hamburg-Eppendorf, Martinistr. 52, 20246 Hamburg, Germany; [u.schumacher@uke.de](mailto:u.schumacher@uke.de) (U.S.)

^5^ Tumor and Breast Center ZeTuP St. Gallen, Rorschacher Strasse 150, 9006 St., Switzerland

^*^ these authors contributed equally

**Reagents and antibodies**

Cisplatin (*cis*-diamminedichloroplatinum (II), 1 mg/ml), gemcitabine, and anisomycin were purchased from NeoCorp (Weilheim, Germany); z-VAD(OMe)-fmk from Enzo Life Sciences (Farmingdale, NY, USA); MTT (3-(4,5-dimethylthiazol-2-yl)-2,5-diphenyltetrazolium bromide) reagent, propidium iodide (PI), and chloroquine from Sigma (Taufkirchen, Germany); annexin-V-FITC from BD Bioscience (San Jose, CA, USA); SP600125 and bafilomycin A1 from LC Laboratories (Woburn, MA, USA). Primary and secondary antibodies used are listed in Table S1.

**Table S1.** List of antibodies used

| Antibodies | Clonality | Source | Cat.-No. | Used  concentration | Manufacturer |
| --- | --- | --- | --- | --- | --- |
| anti-α-Tubulin | mAb | mouse | T5168 | 1:5000 | Sigma-Aldrich |
| anti-β-Actin-HRP | pAb | goat | sc-1616 | 1:10000 | Santa Cruz |
| anti-Bad | mAb | rabbit | #9239 | 1:1000 | Cell Signaling |
| anti-Bax | mAb | rabbit | #5023 | 1:1000 | Cell Signaling |
| anti-Bcl-2 | pAb | rabbit | #2876 | 1:1000 | Cell Signaling |
| anti-cleaved Caspase-3 | mAb | rabbit | #9664 | 1:1000 | Cell Signaling |
| anti-ERK | mAb | mouse | #9107 | 1:2000 | Cell Signaling |
| anti-JNK | mAb | rabbit | #9258 | 1:1000 | Cell Signaling |
| anti-LC3B-I/II | pAb | rabbit | #2775 | 1:1000 | Cell Signaling |
| anti-p21^Waf1/Cip1^ | mAb | rabbit | #2947 | 1:1000 | Cell Signaling |
| anti-p38 | mAb | rabbit | #9212 | 1:1000 | Cell Signaling |
| anti-PAK1 | pAb | rabbit | #2602 | 1:1000 | Cell Signaling |
| anti-PARP | pAb | rabbit | #9542 | 1:1000 | Cell Signaling |
| anti-phospho-ERK | mAb | rabbit | #4377 | 1:1000 | Cell Signaling |
| anti-phospho-JNK | mAb | rabbit | #4668 | 1:1000 | Cell Signaling |
| anti-phospho-p38 | mAb | rabbit | #4511 | 1:1000 | Cell Signaling |
| anti-SQSTM/p62 | pAb | rabbit | #5114 | 1:1000 | Cell Signaling |
| anti-Survivin | pAb | rabbit | NB500-201 | 1:1000 | Novus |
| anti-rabbit IgG-Allexa Fluor^®^ 488 |  | goat | #4412 | 1:1000 | Cell Signaling |
| anti-goat IgG-HRP |  | rabbit | #31433 | 1:10000 | Thermo Scientific |
| anti-mouse IgG-HRP |  | sheep | NXA931 | 1:10000 | GE Healthcare |
| anti-rabbit IgG-HRP |  | goat | #7074 | 1:5000 | Cell Signaling |

Cell culture conditions

Cells were incubated at 37°C in a humidified atmosphere with 5% (v/v) CO_2_. Cells were continuously kept in culture for a maximum of 3 months, and were routinely inspected microscopically for stable phenotype and regularly checked for contamination with mycoplasma.

RT112, RT4, TCC(sup), T-24, and 486p cells were cultured in 10% FBS/RPMI medium (RPMI medium supplemented with Glutamax^TM^-I (Invitrogen, Paisley, UK) containing 10% fetal bovine serum (FBS, Invitrogen) and 1% penicillin/streptomycin (Invitrogen)). HT-1197 cells were cultured in 10% FBS/DMEM medium (RPMI medium supplemented with Glutamax^TM^-I containing 10% FBS, 1% penicillin/streptomycin, and 1 mM sodium pyruvate (Invitrogen)). HT-1197 cells were cultured in 10% FBS/DMEM medium (DMEM medium supplemented with Glutamax^TM^-I (Invitrogen) containing 10% FBS and 1% penicillin/streptomycin (Invitrogen)).
